# Physical Activity Evaluation Using a Voice Recognition App: Development and Validation Study

**DOI:** 10.2196/19088

**Published:** 2021-01-21

**Authors:** Hideyuki Namba

**Affiliations:** 1 Physical Education Lab. College of Science and Technology Nihon University Chiba Japan

**Keywords:** voice recognition, smartphone, physical activity, accelerometer, application

## Abstract

**Background:**

Historically, the evaluation of physical activity has involved a variety of methods such as the use of questionnaires, accelerometers, behavior records, and global positioning systems, each according to the purpose of the evaluation. The use of web-based physical activity evaluation systems has been proposed as an easy method for collecting physical activity data. Voice recognition technology not only eliminates the need for questionnaires during physical activity evaluation but also enables users to record their behavior without physically touching electronic devices. The use of a web-based voice recognition system might be an effective way to record physical activity and behavior.

**Objective:**

The purpose of this study was to develop a physical activity evaluation app to record behavior using voice recognition technology and to examine the app’s validity by comparing data obtained using both the app and an accelerometer simultaneously.

**Methods:**

A total of 20 participants (14 men, 6 women; mean age 19.1 years, SD 0.9) wore a 3-axis accelerometer and inputted behavioral data into their smartphones for a period of 7 days. We developed a behavior-recording system with a voice recognition function using a voice recognition application programming interface. The exercise intensity was determined from the text data obtained by the voice recognition program. The measure of intensity was metabolic equivalents (METs).

**Results:**

From the voice input data of the participants, 601 text-converted data could be confirmed, of which 471 (78.4%) could be automatically converted into behavioral words. In the time-matched analysis, the mean daily METs values measured by the app and the accelerometer were 1.64 (SD 0.20) and 1.63 (SD 0.20), respectively, between which there was no significant difference (*P*=.57). There was a significant correlation between the average METs obtained from the voice recognition app and the accelerometer in the time-matched analysis (*r*=0.830, *P*<.001). In the Bland-Altman plot for METs measured by the voice recognition app as compared with METs measured by accelerometer, the mean difference between the two methods was very small (0.02 METs), with 95% limits of agreement from –0.26 to 0.22 METs between the two methods.

**Conclusions:**

The average METs value measured by the voice recognition app was consistent with that measured by the 3-axis accelerometer and, thus, the data gathered by the two measurement methods showed a high correlation. The voice recognition method also demonstrated the ability of the system to measure the physical activity of a large number of people at the same time with less burden on the participants. Although there were still issues regarding the improvement of automatic text data classification technology and user input compliance, this research proposes a new method for evaluating physical activity using voice recognition technology.

## Introduction

There has been remarkable progress in wearable devices (the collective name for information devices that are worn and carried) in recent years. Particularly, devices that objectively record lifelog data, such as physical activity and sleep, have attracted attention [1]. Many companies have developed smartphones and wristwatch-type behavior-recording sensors. Wearable devices such as these are expected to contribute to the promotion of physical activity for many people [2]. According to the global market forecast for mobile health solutions (2015-2022), the global mobile health market is expected to reach $90.4 billion in 2022 from $21.1 billion in 2017 [3]. The global health market, along with the Internet of Things and big data analysis, has become an important part of Japan's growth strategy for the fourth industrial revolution [4]. Despite the fact that deficient physical activity is the third leading cause of death [5], physical activity in Japan has reportedly decreased slightly over the past 10 years [6].

The questionnaire method has excellent cost performance and is suitable for large-scale physical evaluation surveys, but it has been pointed out that there is a problem with its validity [7]. An epidemiological study [8] used a smartphone app (the Argus app) to demonstrate that step count is negatively associated with obesity. However, most wearable devices with built-in accelerometers have been shown to underestimate the physical activity energy expenditure value of the doubly labeled water [9]. Although accelerometers are highly useful, data bridging might be necessary in epidemiologic studies due to underestimation (depending on the wearable device used to measure energy expenditure). In a previous study [10], we developed and validated a physical activity measurement system based on a 24-hour activity recording method, to be used as an accessible physical activity evaluation system. The accuracy of the energy expenditure measurement was determined using the doubly labeled water method. The average energy expenditure measurement value and the average value of the doubly labeled water nearly matched, and the two were very highly associated [10]. In another study [11], we collected data from thousands of people using this physical activity measurement system and conducted epidemiological studies such as regional comparisons. We found that our system can evaluate physical activity in a large number of people simultaneously, with substantial accuracy and at a low cost. However, the users are tasked with inputting information about the activities in which they have engaged every 15 minutes for the 24 hours in a day. As a result, users are required to make an effort and input time while stopping physical activity.

To address these problems, we attempted, in this study, to develop a behavior-recording method using voice recognition. Voice recognition technology enables the recognition and translation of verbal information into text, which can then be used in automated data processing systems. Using voice recognition technology enables behavior recording without requiring users to touch electronic devices. Information and communication technology–based telehealth programs with voice recognition technology show the potential to improve the health of patients with chronic heart failure by self-care management behaviors [12]. With the improvement of voice recognition technology accuracy, such programs are used in the medical field to enhance the adherence of health behavior of patients with chronic diseases [13,14]. The possibility of a voice-based mobile nutrition monitoring system that uses voice processing has been reported [15]. However, we do not know of any research that has, to date, used voice recognition technology to evaluate physical activity. Because it has been shown that physical activity can be estimated with high accuracy from 33 types [16] and 66 types [10] of behavior records, we hypothesized that physical activity could be evaluated by using voice input that could then be converted into behavioral text.

If this study could prove the validity of physical activity evaluation using a behavior recording method that relies on voice recognition technology, it might be possible to evaluate physical activity more simply in the future. This study may improve the validity of physical activity evaluation and the usability of voice recognition devices.

The purpose of this study was to develop a physical activity evaluation app that records behavior using voice recognition and to examine the validity of the method by using the app and accelerometer at the same time.

## Methods

### Participants

A total of 20 healthy students (14 men and 6 women) with an average age of 19.1 (SD 0.9) years participated in the study. The developed voice recognition app was downloaded to their smartphones and a 1-week behavior record was inputted. We explained the purpose of the survey to the participants and the voluntary nature of their participation. We explained that the privacy and anonymity of the participants would be strictly observed, confirmed that there were no related health concerns, and obtained consent. This research project was approved by the Nihon University College of Sports Science for Research Ethics Review (2019-012).

### Voice Recognition App Development

**Figure 1:**
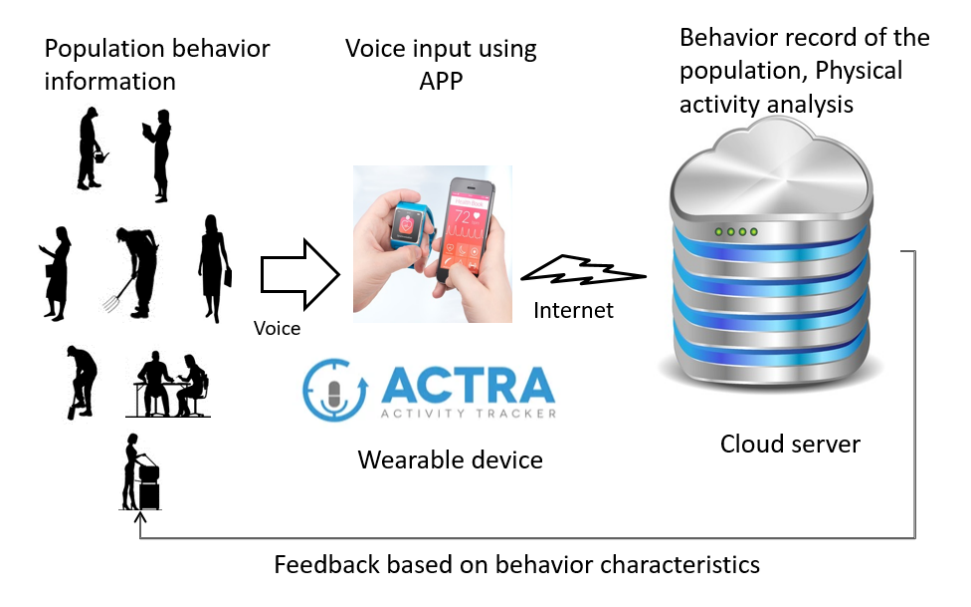
A behavior-recording system with a voice input app.

**Figure 2:**
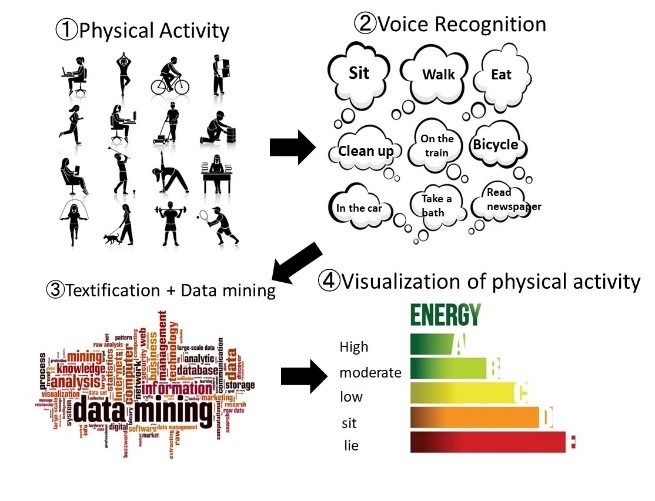
Conceptual view of the visualization of physical activity from the voice input.

We developed a behavior-recording system with a voice recognition function (Figures 1 and 2) using a voice recognition application programming interface. In this research, we commissioned the development of a voice recognition app called ACTRA (Yonefu International Inc, Fukuoka) using the SIRI application program interface (Apple Inc, California). SIRI's speech recognition technology uses an acoustic model and a language model to convert speech data into text via a server. Attempts have been made to utilize various speech recognition technologies, including SIRI, in the field of health medicine [17-19]. The basic structure of our physical activity evaluation system is based on the Web-Based Physical Activity Measurement System [10] and Web-Based Physical Activity Records [11] developed with reference to a simplified physical activity record [16]. By accumulating data in a cloud server and analyzing big data, we were able to analyze behavior patterns, which enabled the categorization of behavior patterns and the estimation of future physical activity from current physical activity patterns.

**Figure 3:**
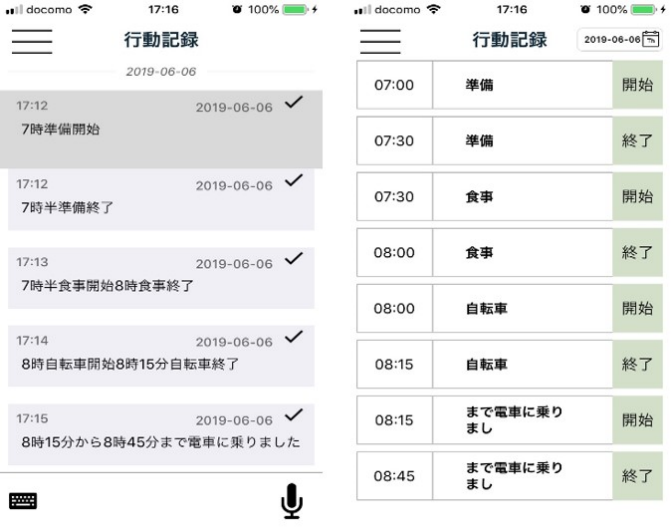
Screen of the developed voice recognition app.

We developed an algorithm that can calculate total energy expenditure based on voice data. To our knowledge, this method is the first to evaluate physical activity and energy expenditure based on a behavioral record generated via self-inputted voice data. Figure 3 shows the screen of the developed voice recognition app. When the user presses the microphone button and tells the smartphone what they are or were doing, their voice is recognized and converted into text data (Figure 3, left). The terms “start,” “end,” “from,” “to,” and “do” were set as key words to separate words and list the activity in time units with the machine learning function (Figure 3, right). Participants could use the device in real time, such as by saying “start now” when they began an activity. They could also look back at the activities completed that day, and input activities as follows: “Starting at △△〇〇 hour □□ minute, ●● hour ▼▼ minute end.” The behavior database is queried from the word, and the “start” and “end” of the trigger word are extracted. Then, the total time of each behavior is extracted, and the activity intensity from the start to the end of the behavior is automatically recorded. During the study, it was difficult to convert the recorded behavior into text completely automatically, and in some cases human judgment was necessary. The exercise intensity was determined from the text data obtained by the voice recognition program as well as from the correspondence table [20] between the behavior and exercise intensity. For each behavior, the product of exercise intensity and time was obtained, and METs time per day was calculated. The average METs per day was obtained by dividing the total METs value per minute by the total activity time.

### Physical Activity Measurement via Accelerometer

Participants wore a 3-axis Active Style Pro accelerometer (HJA-750C, Omron Healthcare, Kyoto) on their lower back for one week as confirmed by the input data of the physical activity measurement system. The accelerometer was considered to have a better correlation with doubly labeled water in the measurement of physical activity energy expenditure than other accelerometers [9]. Participants were instructed to always wear the unit except for during sleep and bathing. The epoch length for processing the data obtained from the accelerometer was set to 10 seconds. The activity intensity (METs) was estimated from the combined acceleration in the vertical, front-back, and left-right directions, and was collected in units of 10 seconds. Activity intensity below the detection threshold, categorized as “a zero count,” was a period in which no activity was detected for more than 60 minutes. This time was considered to correlate to periods when the participant was not wearing the device (nonwearing time); the wearing time was determined by subtracting the nonwearing time from 24 hours. Datasets for days with daily wearing times of 10 hours or more were analyzed. To calculate the 24-hour average METs, 0.9 METs were calculated without measurement data.

### Time-Matched Analysis of Both Voice Recognition and Accelerometer Methods

The advantage of using acceleration is that it has high temporal resolution; voice recognition can also be analyzed every minute. Therefore, we performed an analysis in which acceleration data was collated with the behavior recording timeline of voice recognition. As a procedure for time matching, the activity intensity obtained from the accelerometer was matched with multiple behavior records obtained from voice input every minute, and the average METs per day was calculated by each method. The time matching was not automated and was done manually.

### Statistical Analysis

Mean METs per day are shown as the mean with the standard deviation. A paired *t* test was performed to compare the average METs values obtained from the voice recognition app and the 3-axis accelerometer. Pearson's correlation coefficient was calculated to examine the correlation between METs obtained from the voice recognition app and the 3-axis accelerometer. The average METs values were calculated for cases where the voice recognition was recorded for 10 hours of activity or more, 14 hours or more, and for time matching analysis. For the values calculated under each analysis condition, the average METs values were compared, and the correlation coefficient was calculated. Using Bland-Altman plots, we related the difference in METs between voice recognition and accelerometer (y axis) to the arithmetic mean of METs for voice recognition and accelerometer (x axis) [21]. Statistical analysis was performed using SPSS version 25 IBM (IBM Corporation, Somers, NY, USA) with an alpha level of less than .05.

## Results

In the voice input data of the subject, 601 text-converted data could be confirmed, of which 471 (78.4%) could be automatically converted into behavioral words. For example, phrases with trigger words such as “study from 9:00 to 12:00,” “start meal at 7:00, end meal at 8:00,” and clear words could be automatically converted. However, automatic conversion was not possible for phrases without trigger words such as “get up at 7:00,” or for long sentences containing objects such as “going home by riding a bicycle.” Therefore, the text data that could not be automatically converted was checked and converted by hand.

Table 1 shows the average METs for the voice recognition app and accelerometer. There were 36 days with more than 10 hours of voice recognition data and 16 days with more than 14 hours of voice recognition data. The average daily METs measured by the accelerometer was 1.47 (SD 0.23) and 1.48 (SD 0.24) compared to 1.56 (SD 0.24) and 1.58 (SD 0.28) when the voice recognition data were analyzed for days with 10 or more hours of activity and 14 or more hours, respectively. Under all data extraction conditions, the average METs value determined by voice recognition was significantly higher than that determined by the accelerometer (≥10 hours *P*=.02, ≥14 hours *P*=.04). In the time-matched analysis, the voice recognition and accelerometer values were 1.64 (SD 0.20) and 1.63 (SD 0.20), respectively, and there was no significant difference (*P*=.57).

**Table 1:** Comparison of average daily METs^a^ measured by voice recognition app and accelerometer.

Dataset	Voice recognition, average METs (SD)	Accelerometer, average METs (SD)	*P* value of the *t* test	Number of analyzed days
Days with 10 or more hours of measured activity	1.56 (0.24)	1.47 (0.23)	0.02	36
Days with 14 or more hours of measured activity	1.58 (0.28)	1.48 (0.24)	0.04	16
Time-matched analysis	1.64 (0.20)	1.63 (0.20)	0.57	36

^a^METs: metabolic equivalents.

**Figure 4:**
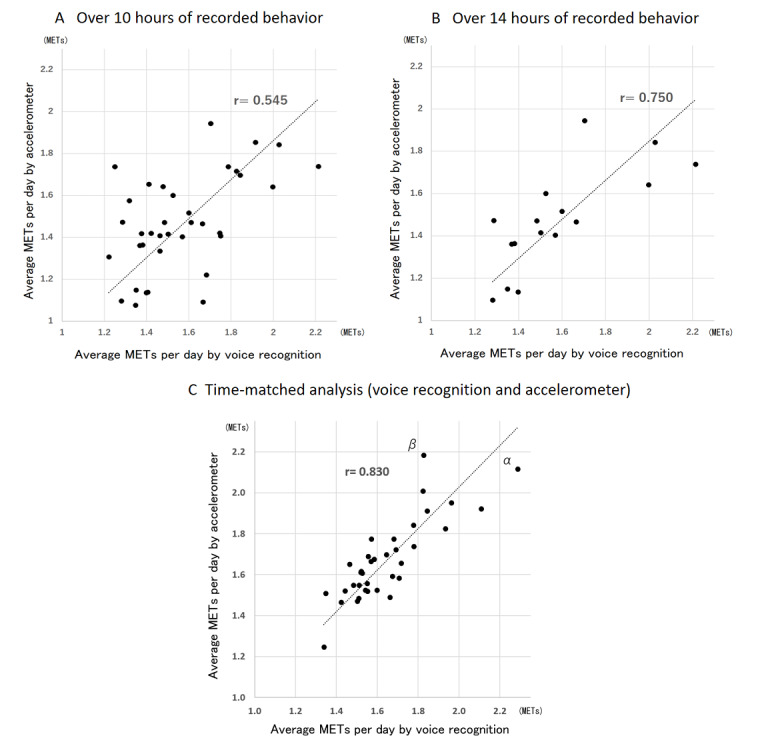
Correlation of average measured METs with voice recognition app and accelerometer. METs: metabolic equivalents.

Figure 4 shows the correlation between the voice recognition data and the average daily METs measured by the accelerometer. Figure 4A shows the data for voice recordings more than 10 hours long, Figure 4B shows the data for recordings that were more than 14 hours long, and Figure 4C shows the date for the time-matched analysis of both voice recognition and accelerometer methods. Under all conditions, the average METs values from voice recognition ranged from 1.2 METs to 2.3 METs. In contrast, the average METs values measured by the accelerometer ranged from 1.0 METs to 2.2 METs. There was a significant correlation between the average METs obtained from voice recognition and that obtained by the accelerometer in the 10 hour dataset, 14 hour dataset, and the time-matched analysis (*r*=0.545, *P*=.02; *r*=0.750, *P*=.008; and *r*=0.830, *P*<.001, respectively).

**Figure 5:**
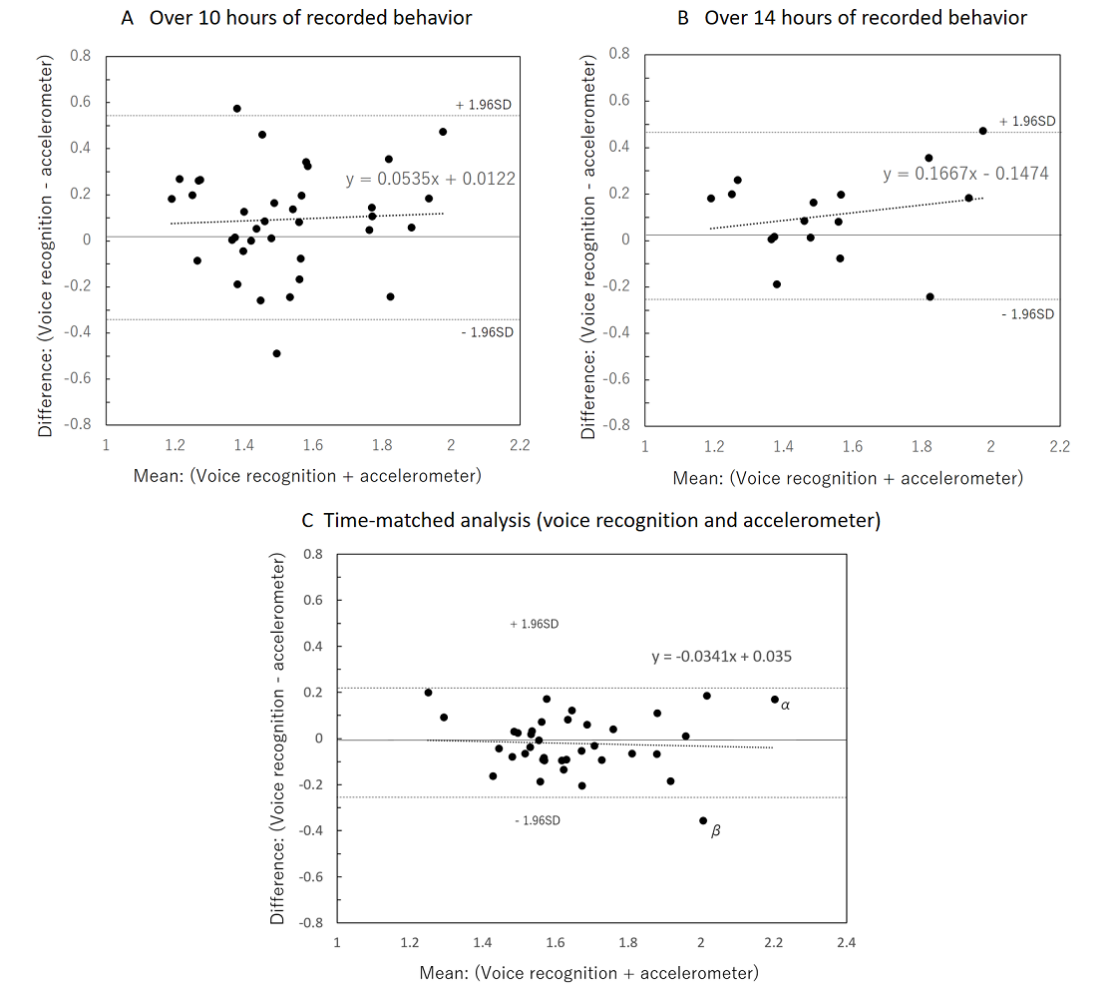
Bland–Altman plot illustrating the difference in average measured METs between the voice recognition app and accelerometer. METs: metabolic equivalents.

Figure 5 shows the Bland-Altman plot for METs as measured by voice recognition compared with METs as measured by the accelerometer. Figure 5A shows the data for voice recordings more than 10 hours long, Figure 5B shows the data for recordings that were more than 14 hours long, and Figure 5C shows the date for the time-matched analysis of both voice recognition and accelerometer methods. The mean difference (Figure 5A) for the voice recognition and the accelerometer was small (0.09 METs), and the limits of agreement were large at 0.44 METs (SD 1.96). The test for trend was not statistically significant. The regression equation was y = 0.054x + 0.012 (*r*=.049, *P*=.78). The mean difference (Figure 5B) between the two methods was small (0.11 METs), and the limits of agreement were large at 0.37 METs (SD 1.96). The test for trend was not statistically significant. The regression equation was y = 0.167x – 0.15 (*r*=.217, *P*=.42). In the Bland-Altman plot (Figure 5C) for METs measured by voice recognition compared with METs measured by the accelerometer, the mean difference between the two methods was very small (0.02 METs), and the limits of agreement were 0.24 METs (SD 1.96). The test for trend was not statistically significant. The regression equation was y = –0.0341x + 0.035 (*r*=–.056, *P*=.74).

**Figure 6:**
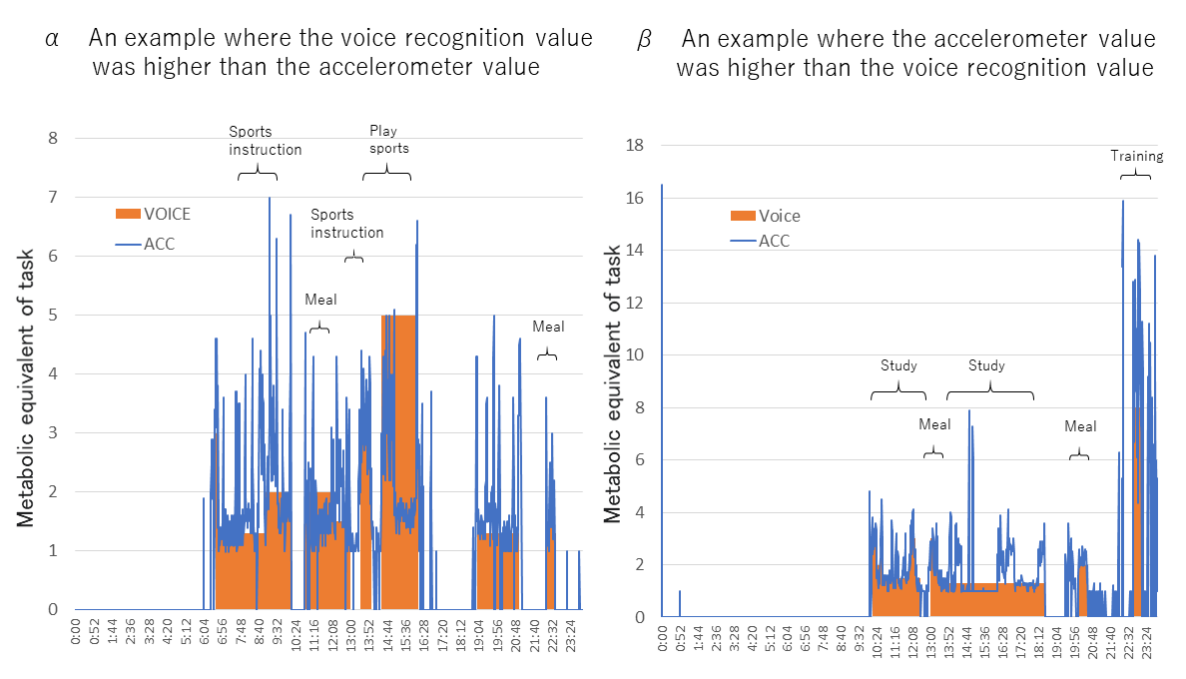
24-hour data for the two high-error examples (α and β in Figure 4C, Figure 5C).

Figure 6 shows two examples in which the error between the METs value measured by voice recognition and the METs value measured value by the accelerometer was large. Figure 6 (α, β) shows the individual data for α and β in Figures 4 and 5. Figure 6α shows that voice recognition app recorded higher METs values than the accelerometer. This was due to the fact that the voice recognition app determined that the game was in progress, while the acceleration data showed a low intensity period. On the other hand, in Figure 6β, the METs values measured by the accelerometer were higher than those measured by the voice recognition app. The cause was judged to be that the voice recognition app recorded the activity as training and set the METs value to 5, whereas, in reality, the activity was accompanied by intense exercise exceeding 10 METs.

## Discussion

### Overview

In this study, we developed a behavior-recording app that uses voice recognition technology and verified the validity of the app using an accelerometer. Participants’ exercise intensity was estimated from the activity recorded using the voice recognition app, and the average daily METs was calculated [10,11]. When the validity of this data was verified using an accelerometer, we found a high correlation between the time-matched analysis of both app- and accelerometer-measured data, and a moderate correlation when the behavior was recorded for both 10 hours or more and 14 hours or more in a single day. Further, the average daily METs value measured by the voice recognition app in time-matched analysis was not significantly higher than that measured by the accelerometer. However, the average daily METs value measured by the voice recognition app was significantly higher than that measured by the accelerometer for both the 10 hour or more dataset and the 14 hour or more dataset. In a previous study [22], a 24-hour behavior recording strategy using a website produced a significantly higher METs value than an accelerometer. Therefore, when estimating energy expenditure via voice recognition recording, the relevance was moderate if it did not time match the acceleration data, but the average might be overestimated.

### Validity of Physical Activity Evaluation Using Voice Recognition

It has been reported that the total energy expenditure reported by an accelerometer underestimates physical activity, even when the gold standard doubly labeled water method is used as a standard [9]. This is because the device is removed during bathing and to charge the device. Therefore, in the present study, the average METs obtained by the voice recognition app might have been slightly higher than that of the accelerometer. In addition, the accelerometer does not count movements that occur in a sitting position or stationary standing position, and acceleration is not measured. Acceleration does not match energy expenditures when riding a bicycle, climbing stairs, or walking on slopes [23]. Also, energy expenditures due to movements of antigravity muscles should be considered [24]. Further, the influence of dietary thermogenesis upon digesting food may be an overlooked factor [7]. Although there is a limit to the physical activity evaluation of the accelerometer, the correlation with physical activity energy expenditure by doubly labeled water has also been shown [9]. In this study, physical activity energy expenditure is not examined because the basal metabolism was not measured, and the average METs was evaluated. Figure 5 (A, B, C) indicates that the error range of the values recorded by voice recognition did not change, regardless of the highest and lowest average METs values. Figure 5 demonstrates no significant correlation between the data in any of the graphs; the error range was shown (0.44 METs (A), 0.37 METs (B), and 0.24 METs (C), 95% confidence interval) regardless of the size of the estimate. In Figure 5B, the regression line of the Brand Altman plot was slightly upward (y = 0.1667X – 0.1474), and the data varied. It might be said that the higher the average METs value, the larger the error. In other words, it could be said that there was a day when the acceleration did not actually move much, even if a high-intensity behavior was inputted by voice. On the contrary, the accelerometer moved well, but, in some cases, it didn’t move much during voice recording.

Therefore, in consideration of the time resolution, which is the merit of voice recognition and accelerometer use, the time of both methods was matched and analyzed. By using this analysis method, the validity was verified by excluding behaviors that cannot be measured by the accelerometer, which is a weak point of the accelerometer. For example, data for periods of bathing, periods of daytime sleep, and instances in which participants forgot to put on the accelerometer were excluded. In Figure 5C, the regression line of the Brand Altman plot was flat (y = –0.0341X – 0.035) and was within the 95% confidence interval, except for one case. Even so, there was a case where there was a large difference between the voice behavior record and the daily average METs recorded by the accelerometer, so Figure 6 shows the details of the α and β data. The graphs of α and β show cases where voice recognition is overestimated and underestimated compared to acceleration data in sports activities. Other than with sports activities, errors occurred when the activity intensity was difficult to understand for long periods, such as when participants rode bicycles or worked part-time jobs.

### Possibility of Behavior Recording by Voice Recognition

In the physical activity measurement system developed previously [10], behavior was reported every 15 minutes. Thus, an activity that was performed for about 10 minutes would have been counted as having taken place for 15 minutes. In this study, we hypothesized that behaviors would be more accurate when recorded via voice recognition because the behavior would be evaluated on a finer timescale. In the time-matched analysis, a high correlation was found between the behavior recorded by voice recognition and the acceleration data, and the mean value also matched, so our hypothesis could be true. However, in the analysis dealing with all the data of one day, there was a difference in the mean METs values taken by the two measurement systems, and the correlation coefficient was moderate. This may be due to participants having experienced difficulty remembering the details of their behaviors, or fatigue related to the task of inputting behaviors sequentially in real time. In recent studies that used the estimation method of physical activity with wearable trackers, data collection compliance and validity were positively correlated, but stricter compliance may have increased the number of excluded data points [25]. It is necessary to correctly calculate the average daily METs, assuming practical application that is useful for dietary guidance and lifestyle improvement.

### Limitations and Future Studies

The limitation of this study is that if participant compliance is not high, the measurement accuracy will decrease. In this study, the average daily METs per person were treated as one dataset. If all inputs were perfect, there would have been a total of 140 days of recording, but the final data set we analyzed included 36 days with 10 or more hours of activity and 16 days with 14 or more hours of activity. Increasing compliance when evaluating physical activity using voice recognition is an important factor for future research. There was a technical problem to convert all voice data automatically and mechanically into exercise intensity. However, this study is the first report to validate the evaluation of physical activity using voice input technology. The advantage of the app developed in this research is that it is possible to record behavior by voice input with just a single touch on the device.

This section describes applied research and practice for the future of this research. The method developed in this study could replace the traditional questionnaire in epidemiological studies and could be used to evaluate the physical activity of many people with less burden on the subjects. Time-matched analysis showed that physical activity could be evaluated with extremely high accuracy using voice recognition technology. The developed app enables a simple and low-cost evaluation of physical activity measurement, which may contribute to disease prevention. Future apps that incorporate deep learning using artificial intelligence may be useful for physical activity evaluation research. Although the details of participants’ behavior are not known from the acceleration data, the voice recognition method might be useful for analyzing many behavior patterns.

### Conclusions

We developed a behavior-recording app using voice recognition and examined its validity using an accelerometer. The system was found to be an effective method for collecting physical activity data and is appropriate for use in epidemiological studies. Although the conversion of voice-to-text data into behavior was not perfect, voice recognition technology is evolving day by day and improvement could be expected. The results of the time-matched analysis showed that physical activity could be measured with high accuracy by voice recognition technology. Participant compliance when using voice input technology is important for ensuring data validity. This research proposes a new method for evaluating physical activity using voice recognition technology.

## Abbreviations

METs: metabolic equivalents
